# Prediction of breast cancer by profiling of urinary RNA metabolites using Support Vector Machine-based feature selection

**DOI:** 10.1186/1471-2407-9-104

**Published:** 2009-04-05

**Authors:** Carsten Henneges, Dino Bullinger, Richard Fux, Natascha Friese, Harald Seeger, Hans Neubauer, Stefan Laufer, Christoph H Gleiter, Matthias Schwab, Andreas Zell, Bernd Kammerer

**Affiliations:** 1 Center for Bioinformatics Tübingen (ZBIT), Sand 1, D-72076 Tübingen, Germany; 2 University Hospital Tübingen, Institute of Pharmacology and Toxicology, Department of Clinical Pharmacology, Otfried-Müller-Str. 45, D-72076 Tübingen, Germany; 3 University Hospital Tübingen, Universitäts-Frauenklinik, Calwerstr. 7, D-72076 Tübingen, Germany; 4 Institute of Pharmacy, Auf der Morgenstelle 8, D-72076 Tübingen, Germany; 5 Dr Margarete Fischer-Bosch Institute of Clinical Pharmacology, Auerbachstr. 112, D-70376 Stuttgart, Germany

## Abstract

**Background:**

Breast cancer belongs to the most frequent and severe cancer types in human. Since excretion of modified nucleosides from increased RNA metabolism has been proposed as a potential target in pathogenesis of breast cancer, the aim of the present study was to elucidate the predictability of breast cancer by means of urinary excreted nucleosides.

**Methods:**

We analyzed urine samples from 85 breast cancer women and respective healthy controls to assess the metabolic profiles of nucleosides by a comprehensive bioinformatic approach. All included nucleosides/ribosylated metabolites were isolated by cis-diol specific affinity chromatography and measured with liquid chromatography ion trap mass spectrometry (LC-ITMS). A valid set of urinary metabolites was selected by exclusion of all candidates with poor linearity and/or reproducibility in the analytical setting. The bioinformatic tool of Oscillating Search Algorithm for Feature Selection (OSAF) was applied to iteratively improve features for training of Support Vector Machines (SVM) to better predict breast cancer.

**Results:**

After identification of 51 nucleosides/ribosylated metabolites in the urine of breast cancer women and/or controls by LC- ITMS coupling, a valid set of 35 candidates was selected for subsequent computational analyses. OSAF resulted in 44 pairwise ratios of metabolite features by iterative optimization. Based on this approach ultimately estimates for sensitivity and specificity of 83.5% and 90.6% were obtained for best prediction of breast cancer. The classification performance was dominated by metabolite pairs with SAH which highlights its importance for RNA methylation in cancer pathogenesis.

**Conclusion:**

Extensive RNA-pathway analysis based on mass spectrometric analysis of metabolites and subsequent bioinformatic feature selection allowed for the identification of significant metabolic features related to breast cancer pathogenesis. The combination of mass spectrometric analysis and subsequent SVM-based feature selection represents a promising tool for the development of a non-invasive prediction system.

## Background

Among all cancer diseases, breast cancer is worldwide the most frequent cause of death for women between 30 and 60 years, responsible for approximately 500,000 casualties per year in 2002 [1]. The treatment of cancer diseases is inherently linked to early stage diagnosis. The determination of tumor markers represents an integral part of clinical therapy concepts. Unfortunately, the established markers of breast cancer (e.g. CA-15-3 and CEA) offer only unsatisfactory prediction accuracy and therefore are not recommended for early diagnosis and therapy surveillance [2].

New technological and biological developments have the potential to increase the likelihood of discovering new biomarker candidates. In the systems biology context, novel targets have been identified on the genome-, transcriptome- and proteome level. Recently, the metabolome, representing the end products of physiological processes, has experienced increasing clinical attention.

Cell proliferation can also be controlled by metabolites in a way similar to direct gene regulation. By triggering concentration-dependent state changes in the expression of transcription factors or induction of epigenetic processes, metabolites are able to influence cancer pathogenesis and therefore may play a critical role during tumor progression.

Modified nucleosides, which are degradation products of the cellular RNA metabolism, are suggested to be important as possible tumor markers. In addition to the primary constituents adenosine, guanosine, uridine and cytidine, series of derived modified analogs are well known. These modifications (e.g. methylation, sulfur/oxygen-substitution, hypermodification) are posttranscriptionally implemented in the polynucleotide macromolecules and are considered to increase efficiency, activity and integrity of RNA function [3]. Currently more than 100 modified structures are known for various RNA types [4].

During RNA turnover, hydrolytic enzymes catabolize polynucleotides to the ribonucleoside level. The common ribonucleosides and corresponding nucleobases can partly be recycled to rebuild intracellular RNA in the salvage pathway. Due to the lack of specific phosphorylases, modified nucleosides cannot enter this recycling passage and therefore are excreted quantitatively as biochemical end products [5]. Any disease or metabolic imbalance affecting RNA turnover consequently results in altered nucleoside excretion patterns, leading to the hypothesis that RNA metabolites may be useful as tumormarkers. Supporting this idea, significantly increased amounts of modified nucleosides were found in urine from patients suffering from breast carcinoma [6], leukemia [7] and lung carcinoma [8].

In terms of analytics, the coupling of liquid-, gas- or capillary liquid chromatography with mass spectrometric techniques like ESI-IT MS [9] and ESI tandem MS [10] has been established as method of choice. In addition, systems such as ESI-TOF MS [11], MALDI-TOF MS [12] and especially FTICR MS [13] are valuable tools for the elucidation of chemical structures.

The aim of our study was to classify patients with breast cancer compared to healthy volunteers, based on LC-MS analysis of urinary nucleosides using machine learning techniques, which extract patterns from data and build predictors. For instance principal component analysis (PCA) is a commonly used method which was applied by Yang et al. for classification of liver cancer patients by means of HPLC-UV analysis. Based on a set of 15 nucleosides, 83% of the tumor patients were correctly classified [14]. Artificial neural network (ANN) analysis of urinary nucleosides was used by Seidel et al. to distinguish between healthy controls and patients suffering from various cancer diseases, yielding a sensitivity of 97% and a specifity of 85% [15], respectively.

Recently the support vector machine (SVM) became increasingly popular due to its kernel approach and high practical robustness. This technique has been applied in various clinical research projects, analyzing tumor-associated variances in the genomic profile [16], in addition to protein expression [17] and metabolic [18] patterns. Modified nucleosides have also been the target for SVM approaches. For example Mao et al. [19] utilized CE-MS measurements of RNA metabolites for classification of bladder cancer patients (sensitivity 90%, specifity 100%), whilst previous work in our research group also revealed the classification potential of modified nucleosides (sensitivity 94%, specifity 86% [20] and sensitivity 88%, specifity 90% [21]).

**Figure 1:**
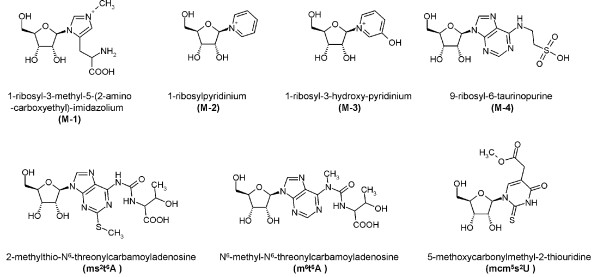
**Some metabolite structures**. Structures of some previously unknown urinary metabolites included in this study. M-4: structure proposal based on combined FT MS and IT MS^n ^analysis. Others: identified in previous works [13].

Whereas clinical metabolomics often analyzes absolute concentration values of a restricted set of metabolites [15,19,21], the present work follows an extended approach. According to the network characteristics in metabolism, we additionally analyzed compounds from pathways, interconnected to cellular RNA catabolism such as histidine metabolism, purine biosynthesis and methionine/polyamine cycle as well as from the nicotinate/nicotinamide metabolism (Figure 1). Furthermore, we used pairwise encoded metabolite ratios in order to assess tumor-associated shifts between substrates in the metabolic flux.

## Methods

### Chemicals

Methanol LiChroSolv, hypergrade, purchased from Merck/VWR (Darmstadt, Germany) was used for liquid chromatography. Water was taken from an in-house double distillation system. All other chemicals used were of analytical grade.

Standard compounds available as reference for HPLC separation and/or compound identification [13] were dihydrouridine (DHU), pseudouridine (Ψ), cytidine (C), pyridine, 3-hydroxypyridine, uridine (U), 3-methylcytidine (m^3^C), 1-ribosyl-4-carbamoyl-5-aminoimidazole (AICA riboside), 1-methyladenosine (m^1^A), 7-methylguanosine (m^7^G), inosine (I), 3-methyluridine (m^3^U), adenylosuccinic acid (phosphorylated analog of N^6^-succinyloadenosine), xanthosine (X), S-adenosylhomocysteine (SAH), 1-methylinosine (m^1^I), 1-methylguanosine (m^1^G), N^4^-acetylcytidine (ac^4^C), N^2^-methylguanosine (m^2^G), N^2^, N^2^,7-trimethylguanosine (m^2,2,7^G), N^2^, N^2^-dimethylguanosine (m^2^_2_G), N^6^-threonylcarbamoyl-adenosine (t^6^A), 5'-deoxy-5'-methyl-thioadenosine (MTA).

All standards were from Sigma (Taufkirchen, Germany) except m^2^_2_G, m^2,2,7^G and t^6^A, obtained from Biolog (Bremen, Germany), 1-methyl-L-histidine, purchased from Calbiochem/Merck (Nottingham, UK) and pyridine from Gruessing (Filsum, Germany). The internal standard isoguanosine was kindly donated by Prof. J.H. Kim of Seoul University, South Korea. Affigel boronate was purchased from Biorad (Richmond, USA).

### Urine samples

**Figure 2:**
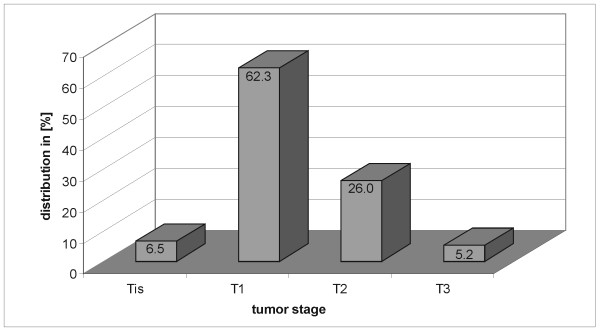
**Tumor stage distribution**. Histogram of the tumor stage distribution. The major fraction of patients had breast cancer in the T1 stadium. The remaining patients were mostly T2 with the exception of 11.7% that divide up into the T3 and the Tis stadium.

**Figure 3:**
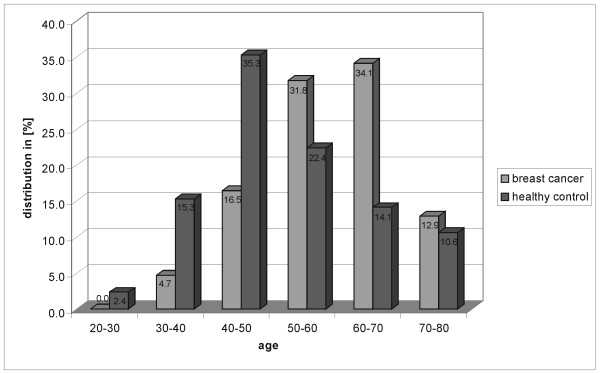
**Age distribution**. Histogram of the age distributions for cancer and control patients.

Spot urine samples were collected from 85 female breast cancer patients (primarily in early tumor stage T1) at the Department of Gynecology and Obstetrics, University Hospital Tuebingen and from 85 female healthy volunteers in a private clinical office (i.e. women accompanying their children to the clinical office). Tumor stage and age distributions are given in figure 2 and 3. The clinical trial has been approved by the local ethics committee of University Hospital Tübingen. In order to minimize possible endo- and exogenous perturbations on the urinary metabolite pattern, we defined precise criteria for patient recruitment. The samples were taken preoperatively and neoadjuvant endocrine therapy, irradiation or chemotherapy were not allowed. Patients taking immunomodulating drugs, antibiotics, mistletoe preparations, virustatics, allopurinol and dipyridamol were not included in this study. Pregnancy, immune mediated diseases, HIV, acute and chronic hepatitis, chronic renal failure, acute infection of the urinary tract as well as the patients' participation in a clinical drug trial were defined as exclusion criteria. All samples were stored at -80°C until extraction.

### Sample preparation

The metabolites were isolated from urine samples by cis-diol specific affinity chromatography with 500 mg affigel boronate per column (column dimensions: 150 × 15 mm). A volume of 1 mL urine was spiked with 50 μL of internal standard solution (0.1 mM isoguanosine in water), mixed with 9 mL ammonium acetate solution (0.25 mM, pH 8.8) and then put on the column following preconditioning with 45 mL ammonium acetate solution (0.25 mM, pH 8.8). Because of the high backpressure from the affigel boronate material, compressed air was applied throughout the extraction procedure to maintain a moderate flow rate at 3–4 mL/min. Ribosylated compounds are bound reversibly and specifically at the 2',3'-cis-diol group. After washing with 10 mL ammonium acetate solution (0.25 mM, pH 8.8) and 4 mL ammonium acetate solution (0.25 mM, pH 8.8)/methanol (9.5:0.5, v/v), elution was carried out with 6 mL methanol/water (2:8, v/v) and 50 mL 0.2 M formic acid in methanol/water (1:1, v/v). The column was reconditioned with 25 mL methanol/water (2:8, v/v) and 45 mL ammonium acetate solution (0.25 mM, pH 8.8) for the next sample. After each second extraction, a blank sample (10 mL ammonium acetate solution (0.25 mM, pH 8.8)) was analogously proceeded to remove impurities from the column and to avoid possible carry-over effects. The solvent from the sample eluate was removed using a rotary evaporator and the residuum was dissolved again in 0.5 mL ammonium formate solution (5 mM, pH 5). A volume of 10 μL was injected into the HPLC-MS system.

### LC-ESI-IT MS

The chromatographic separation of the urinary metabolites was performed on an Agilent 1100 Series HPLC system (Agilent, Waldbronn, Germany) consisting of a Solvent Degasser (G 1379 A), a binary capillary pump (G 1389), an autosampler (G 1313 A), a column oven operated at 25°C (G 1316 A) and a DAD (G 1315 B). The chromatographic system consisted of a Merck LiChroCART Superspher 100 RP-18 endcapped column (125 × 2.0 mm i.d., 4 μm (Merck, Darmstadt, Germany)) and a solvent system of 5 mM ammonium formate buffer, pH 5.0, and methanol-water (3:2, v:v + 0.1% formic acid) at a flow rate of 125 μL/min [9]. The LC-system was coupled to an Esquire HCT-Ion trap mass spectrometer (Bruker Daltonics, Bremen, Germany), equipped with an ESI source and operated in positive ion detection mode.

The capillary voltage was set to 4 kV, the drying gas temperature in the electrospray source was set to 350°C, the nebulizer gas was set to 45 psi and the drying gas to 9.0 L/min. The data were acquired in standard enhanced scan mode (8,100 *m/z *per second) over a mass range of *m/z *200–600 via Bruker EsquireControl version 5.1. For post processing, Bruker DataAnalysis version 3.1 was used.

### Integration procedure

Semiquantitative concentration values were obtained via integration of Extracted Ion Chromatograms (EIC). Due to significant alkali affinity of certain analyzed metabolites, we generally summarize the corresponding [MH]^+^, [MNa]^+ ^and [MK]^+ ^traces. The EICs were processed with a Gauss function smoothing algorithm contained in the DataAnalysis software. For analytical and physiological normalization, the integrated peak areas were related to the internal standard and the urinary creatinine level (in mg/dl):(1)

### Bioinformatic data analysis

For bioinformatic feature selection we encoded pairwise combinations of semiquantitative concentrations of the 35 included metabolites (x, y). This resulted in 35 × 34 = 1190 encodings per sample. We used the encoding function(2)

and defined the case , when *y *= 0 was not detected.

Two problems are solved using this encoding, which should be considered as a normalization step. Firstly, a consistent value for the case where a value was below the detection threshold is obtained, and secondly, this encoding maps *e*(*x*, *y*) and *e*(*y*, *x*) onto different codomain ranges conserving argument order information. For more information [see Additional file 1, 2 and 3].

Next, we applied Linear Discriminant Analysis (LDA) [22] to visualize our encoding. As in the case of Principal Component Analysis (PCA) a linear model is used to visualize the data. In contrast to PCA the aim of LDA is to find a linear model that maximally separates the classes on a straight line.

**Figure 4:**
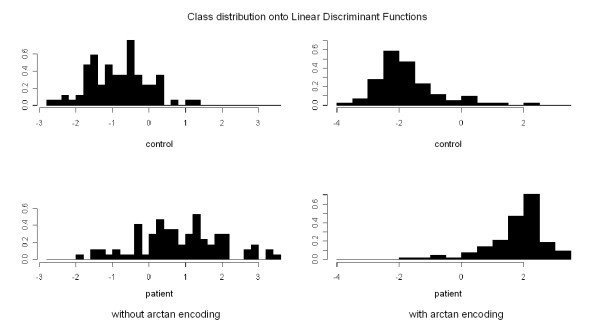
**LDA analysis**. Projection of the class distribution onto a straight line by Linear Discriminant Analysis for the discrete and the arctan-encoded metabolite ratios. As can be seen, the pairwise-encoding offers a better partitioning of cancer and healthy collectives by a linear model than the sole concentration features.

To compare the encodings, we computed the LDA projections to visualize the data set with and without arctan-encoding (see figure 4). Because of the risk of overfitting using more than 1190 features, nonparametric feature selection is needed to reduce the number of features used for prediction.

Therefore we used the Oscillating Search Algorithm for Feature Selection (OSAF) [23] in combination with a SVM to select a reduced set of optimized features for classification. The OSAF wrapper method applies an efficient strategy to select combinations from the power set of features and uses the SVM as a black box to assess the information content [24].

Our implementation operates in up- and down-swing phases which are based on Sequential Forward Selection (SFS) and Sequential Backward Selection (SBS). The SFS greedily includes the feature, which maximally improves the prediction error, while SBS removes the feature which minimally reduces the error.

Having selected a feature set, the algorithm uses the SVM to train a predictor for estimation of the generalization performance. We evaluated the 10-fold cross validated (CV) Matthews Correlation Coefficient (MCC) as measure.

Given the true positives (TP), the true negatives (TN), the false positives (FP) and the false negatives (FN), the MCC is computed as(3)

This results in a value between +1.0 for perfect predictions and -1.0 for maximal false predictions.

Furthermore, we computed the Leave-one-out (LOO) estimate, which is an almost unbiased estimate for the true generalization error [25].

During each evaluation of a feature set, the SVM model parameters were chosen by grid search. We used a modified implementation of LibSVM [26] that reports all statistics needed for the computation of the MCC, together with an OSAF wrapper written in Perl. The LDA analysis was performed in R and the mutual information below was computed using Matlab.

To remove redundancy in the list of features, we classified the selected features into tumor and non-tumor relevant according to current literature knowledge. Then we applied SBS to remove all features which had no impact on the MCC and where not tumor relevant.

To visualize the importance of each selected feature, we computed the *mutual information *[22], defined as(4)

This value represents a quantity which measures the mutual dependence between two variables (here class label and metabolite encodings). Although prediction performance is obtained from a complex set of variables, even those variables with small information content may be essential in combination with others (see [24]).

## Results

### Generating a valid metabolic profile

Based on a set of 51 detectable urinary cis-diol metabolites in the applied sample volume of 1 mL urine, we first attempted to define a valid metabolic profile. To this end, two main criteria were established for the inclusion of compounds. First, the respective metabolite should meet the analytical criteria of linearity and reproducibility. Second, the biochemical origin of the compound should constitute a possible tumor-associated background.

In this manner, 16 compounds were excluded due to poor linearity/reproducibility and/or missing pathophysiological relevance. In the latter case, we eliminated potential secondary metabolites from endosymbiontic bacteria, metabolites influenced by nutrition [13] and compounds originated or influenced in sample preparation. The resulting metabolic profile for SVM training is shown in Table 1.

**Table 1:** Included metabolites

**No**.	[MH^+^]	RT	Metabolite	R^2^	RSD [%]	Symbol	Metabolic pathway^#^
**1**	302	3.0	1-ribosyl-3-methyl-5-(2-aminocarboxyethyl)-imidazolium	0.9875	7.4	**M-1**	(H)
**2**	247	4.3	Dihydrouridine	0.9855	6.0	**DHU**	R
**3**	245	4.5	Pseudouridine	0.9889	3.3	**Ψ**	R
**4**	212	5.0	1-ribosyl-pyridinium	0.9771	5.7	**M-2**	(N)
**5**	244	6.5	Cytidine	0.9815	12.0	**C**	R
**6**	346	7.0	3-(3-aminocarboxypropyl)-uridine	0.9957	3.5	**acp^3^U**	R
**7**	302	7.0	5-carbamoylmethyluridine	0.9977	4.0	**ncm^5^U**	R
**8**	228	7.5	1-ribosyl-3-hydroxy-pyridinium	0.9932	2.1	**M-3**	(N)
**9**	245	8.5	Uridine	0.9981	8.4	**U**	R
**10**	258	9.5	3-methylcytidine	0.9787	2.7	**m^3^C**	R
**11**	259	10.5	1-ribosyl-4-carbamoyl-5-amino-imidazole	0.9942	6.0	**AICA riboside**	N
**12**	282	12.0	1-methyladenosine	0.9949	3.1	**m^1^A**	R
**13**	271	14.0	1-ribosyl-5-carbamoyl-2-oxo-pyridine	0.9903	3.7	**2,5-PCNR**	N
**14**	298	17.5	7-methylguanosine	0.9727	10.2	**m^7^G**	R
**15**	269	18.0	Inosine	0.9911	3.0	**I**	R
**16**	271	20.5	1-ribosyl-3-carbamoyl-4-oxo-pyridine	0.9955	2.1	**3,4-PCNR**	N
**17**	296	21.5	1, N^6^-dimethyladenosine	0.9959	2.9	**m^6^_1_A**	R
**18**	259	23.5	3-methyluridine	0.9972	3.0	**m^3^U**	R
**19**	384	24.5	N^6^-succinyloadenosine	0.9568	11.3	**N^6^-SAR**	P
**20**	285	25.0	Xanthosine	0.9974	4.0	**X**	R
**21**	385	27.5	S-adenosylhomocysteine	0.9926	4.4	**SAH**	MP
**22**	283	29.0	1-methylinosine	0.9969	3.8	**m^1^I**	R
**23**	298	31.0	1-methylguanosine	0.9920	4.5	**m^1^G**	R
**24**	293	32.0	?	0.9918	14.9	**293**	?
**25**	286	32.5	N^4^-acetylcytidine	0.9934	7.6	**ac^4^C**	R
**26**	298	33.5	2-methylguanosine	0.9979	8.2	**m^2^G**	R
**27**	376	34.5	9-ribosyl-6-taurinopurine	0.9974	6.2	**M-4**	(R)
**28**	398	40.0	2-methylthio-N^6^-(cis-hydroxyisopentenyl)-adenosine	0.9983	4.5	**ms^2^io^6^A**	R
**29**	326	41.0	N^2^, N^2^,7-trimethylguanosine	0.9911	2.7	**m^2,2,7^G**	R
**30**	312	41.5	N^2^, N^2^-dimethylguanosine	0.9925	4.7	**m^2^_2_G**	R
**31**	333	42.5	5-methoxycarbonylmethyl-2-thiouridine	0.9981	4.6	**mcm^5^s^2^U**	R
**32**	413	46.5	N^6^-threonylcarbamoyladenosine	0.9963	3.5	**t^6^A**	R
**33**	298	48.0	5'-deoxy-5'-methyl-thioadenosine	0.9987	13.3	**MTA**	MP
**34**	427	49.5	N^6^-methyl-N^6^-threonylcarbamoyladenosine	0.9920	5.5	**m^6^t^6^A**	R
**35**	459	50.0	2-methylthio-N^6^-threonylcarbamoyladenosine	0.9887	3.9	**ms^2^t^6^A**	R

Set of included ribosylated metabolites for bioinformatic data evaluation.

^# ^R: RNA metabolism, H: histidine metabolism, N: nicotinate/nicotinamide metabolism,

MP: methionine/polyamin cycle, P: purine biosynthesis, ?: unknown. Abbreviations in parenthesis: pathway proposal, RT: retention time. (Structures M-1 to M-4 see Figure 1.)

#### Proof of reproducibility

For proof of reproducibility, 10 mL of a spot urine sample were spiked with 500 μL internal standard (0.1 mM isoguanosine in water). The obtained solution was separated in ten aliquots of 1 mL. Each aliquot was mixed with 9 mL ammonium acetate solution (0.25 mM, pH 8.8) to give a sample volume of 10 mL, vortexed and proceeded as described in sections "extraction procedure" and "integration procedure". The obtained values for reproducibility are shown in Table 1. A compound was considered to be reproducible for RSD values ≤ 15%.

#### Proof of linearity

For proof of linearity, two different spot urine samples were separated in specimens of 0.25 mL, 0.5 mL, 1 mL, 2 mL and 4 mL. Each sample was spiked with 50 μL internal standard and mixed with 9 mL ammonium acetate solution (0.25 mM, pH 8.8) to give a sample volume of 10 mL. The obtained solutions were proceeded as described. The obtained values are shown in Table 1. Linearity was considered for regression coefficients ≥ 0.95.

### Feature selection with best classification performance

As can be seen in figure 4 both collectives (cancer/healthy) are clearly separable using the arctan-encoding, while the usage of the semiquantiative concentrations yields a poor separation performance. Therefore a learning algorithm can construct better separating models on the transformed data using the arctan-encoding than on the raw feature encoding.

**Figure 5:**
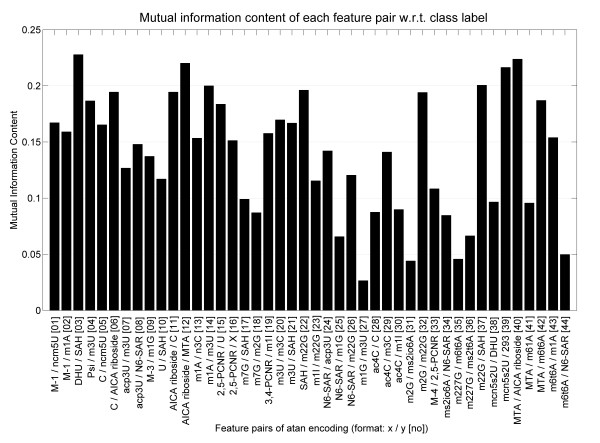
**Mutual Information Content**. This figure shows the mutual information content of the selected metabolite ratios. On the x-coordinate all pairwise encoded features are listed with their indexes in table 2 in brackets.

The application of the OSAF yielded a set of 59 feature combinations with best classification performance. The successive pruning step with SBS left a set of 44 mainly pathophysiological relevant feature combinations, without degrading classification performance (Table 2). Final performance was a sensitivity of 83.5% and a specifity of 90.6% with a p-value << 0.05 (Two-sided Fisher's exact test, Table 3) for 10-fold cross validation. The leave-one-out validation yielded 83.5% sensitivity and a specifity of 85.9% also having p-value << 0.05. Figure 5 shows the mutual information of the selected combination. In comparison to prior work [20] the mutual information identifies more informative features that are obtained by using a pairwise-encoding.

**Table 2:** Selected feature set

**No**.	encoding*	metabolite ratio	**No**.	encoding	metabolite ratio
**1**	arctan (1/7)*	M-1/ncm^5^U	**23**	arctan (20/3)	X/ψ
**2**	arctan (1/12)	M-1/m^1^A	**24**	arctan (20/35)	X/ms^2^t^6^A
**3**	arctan (2/21)	DHU/SAH	**25**	arctan (21/30)	SAH/m^2^_2_G
**4**	arctan (3/18)	Ψ/m^3^U	**26**	arctan (22/30)	m^1^I/m^2^_2_G
**5**	arctan (5/7)	C/ncm^5^U	**27**	arctan (23/18)	m^1^G/m^3^U
**6**	arctan (5/11)	C/AICA riboside	**28**	arctan (25/5)	ac^4^C/C
**7**	arctan (6/18)	acp^3^U/m^3^U	**29**	arctan (25/10)	ac^4^C/m^3^C
**8**	arctan (6/19)	acp^3^U/N^6^-SAR	**30**	arctan (25/22)	ac^4^C/m^1^I
**9**	arctan (8/23)	M-3/m^1^G	**31**	arctan (26/28)	m^2^G/ms^2^io^6^A
**10**	arctan (9/21)	U/SAH	**32**	arctan (26/30)	m^2^G/m^2^_2_G
**11**	arctan (11/5)	AICA riboside/C	**33**	arctan (27/13)	M-4/2,5-PCNR
**12**	arctan (11/33)	AICA riboside/MTA	**34**	arctan (28/19)	ms^2^io^6^A/N^6^-SAR
**13**	arctan (12/10)	m^1^A/m^3^C	**35**	arctan (29/34)	m^2,2,7^G/m^6^t^6^A
**14**	arctan (12/18)	m^1^A/m^3^U	**36**	arctan (29/35)	m^2,2,7^G/ms^2^t^6^A
**15**	arctan (13/9)	2,5-PCNR/U	**37**	arctan (30/21)	m^2^_2_G/SAH
**16**	arctan (13/20)	2,5-PCNR/X	**38**	arctan (31/2)	mcm^5^s^2^U/DHU
**17**	arctan (14/21)	m^7^G/SAH	**39**	arctan (31/24)	mcm^5^s^2^U/293
**18**	arctan (14/26)	m^7^G/m^2^G	**40**	arctan (33/11)	MTA/AICA riboside
**19**	arctan (16/22)	3,4-PCNR/m^1^I	**41**	arctan (33/17)	MTA/m^6^_1_A
**20**	arctan (18/10)	m^3^U/m^3^C	**42**	arctan (33/34)	MTA/m^6^t^6^A
**21**	arctan (18/21)	m^3^U/SAH	**43**	arctan (34/12)	m^6^t^6^A/m^1^A
**22**	arctan (19/30)	N^6^-SAR/m^2^_2_G	**44**	arctan (34/19)	m^6^t^6^A/N^6^-SAR

Feature set for best classification performance.

*Numbering of arctan encoding combinations consistent with table 1. For metabolite abbreviations, please refer to Table 1.

**Table 3:** Generalization performance

Validation	Sensitivity [%]	Specifity [%]	MCC*	TP	FN	TN	FP	p-value
CV (10 fold)	83.5	90.6	0.743	71	14	77	8	2.2 × 10^-16^
LOO	83.5	85.9	0.694	71	14	73	12	2.2 × 10^-16^

Prediction results for the best obtained feature selection. The p-value was computed by the Fishers' Exact Test.

* for abbreviations refer to main text.

## Discussion

The obtained feature selection reflects characteristic, tumor-associated shifts in the analyzed metabolite patterns.

The action of methyltransferases plays a key role in the aberrant RNA metabolism in tumor genesis [27]. In this context, the selected feature combinations of methylated nucleosides No. 13 (m^1^A/m^3^C), 15 (m^1^A/m^3^U), 20 (m^3^U/m^3^C), 26 (m^1^I/m^2^_2_G), 27 (m^1^G/m^3^U), 35 (m^2,2,7^G/m^6^t^6^A) and 36 (m^2,2,7^G/ms^2^t^6^A) show pathophysiologically motivated pattern shifts. Tsutsui et al. already reported on significant alterations in the ratios of the monophosphorylated, methylated nucleosides m^6^A, m^5^C, m^2^G and m^2^_2_G from tRNA in normal hepatocytes and Novikoff-Hepatoma cells [28]. Changes in the enzyme specifity, resulting in an enlarged set of possible modification sites in the polynucleotide molecule, were postulated as biochemical background. Analogous alterations in the methylation capacity have also been reported in breast cancer [29]. The observed shifts in the excretion ratios of certain methylated nucleosides can be generally traced back to this phenomenon.

**Figure 6:**
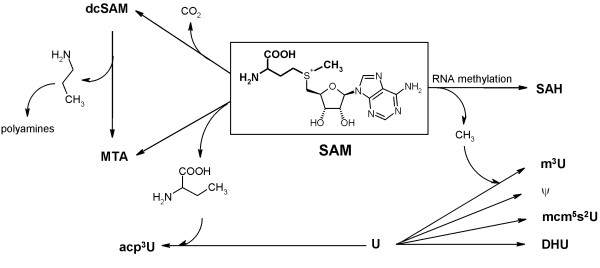
**Connectivity map**. Connectivity map of SAM and related metabolites.

A metabolic pathway with considerable classification potential was found to be the methionine-/polyamine cycle. Striking analogies have been found to our previous projects, dealing with metabolic profiling in cell culture supernatants of breast cancer cell line MCF-7. In this work, characteristic tumor-associated alterations in the methionine/polyamine cycle had been observed for the excretion behavior of the corresponding degradation products [30]. In particular, these were metabolites from the ubiquitous enzymatic co-substrate SAM. Figure 6 shows a connectivity map of the corresponding biochemical pathways.

The ribosyl-conjugated methionine scaffold of SAM provides functional groups for various enzymatic reactions. The biosynthesis of the modified uridine derivative proceeds via selective transfer of the carboxyaminopropyl moiety on uridine positions in the RNA molecule [31]. In this context, the feature combination 10 (U/SAH) is of great importance. The high information content in the classification process is probably based on alterations in the competing reaction pathways SAM → SAH and SAM → U → acp^3^U. In cancer diseases, the elevated cellular methylation capacities lead to higher synthesis and thus excretion of SAH, consequently resulting in altered SAH/U ratios. This presumption is supported by the fact that ratio No. 3 (DHU/SAH) is also differing between breast cancer patients and healthy control subjects. DHU is a uridine derivative, modified through enzymatic reduction of uridine.

The most characteristic indication for tumor-associated alterations in the reaction of SAM-induced carboxyaminopropyltransfer and SAM-induced methyltransfer is reflected by feature combination 7 (acp^3^U/m^3^U). Both modified nucleosides represent the primary metabolites of uridine in the mentioned reaction pathways and contribute to the resulting classification performance of the SVM.

Distinctive metabolite ratios within the sets of modified uridines such as No. 4 (Ψ/m^3^U) and No. 38 (mcm^5^s^2^U/DHU), adenosines (No. 43, m^6^t^6^A/m^1^A) and cytidines (No. 29, ac^4^C/m^3^C) were selected in the course of the performed feature selection due to their high information content. Alterations in the concentration ratios within one nucleoside group can be attributed to tumor-associated changes in expression and activities of the involved modifying enzyme systems.

The deregulation of SAM-induced methyltransfer reactions in tumor genesis is reflected by three additional feature combinations No. 17 (m^7^G/SAH), 21 (m^3^U/SAH) and 37 (m^2^_2_G/SAH). The methylated nucleosides m^7^G, m^3^U and m^2^_2_G are posttranscriptionally synthesized via transfer of the activated SAM methyl function on defined positions in the polynucleotide molecules. The SAM cosubstrate involved is converted to SAH. As a consequence, the elevated methylation capacities in tumor cells result in higher levels of methylated nucleosides and thus an increased degradation of SAM yielding SAH. The latter is known as a potent inhibitor of methyltransferases [32]. An elevated level of excretion has already been observed in our studies on metabolite excretion in cell culture supernatants of tumor cell line MCF-7 compared to breast epithelial cell line MCF-10A [30]. As a main conclusion, tumor cells most likely avoid the aforementioned inhibitory effects by active excretion of surplus SAH, resulting in ratio shifts to methylated nucleosides.

In this context, the feature combinations No. 41 (MTA/m^6^_1_A) and 42 (MTA/m^6^t^6^A) should also be mentioned. MTA is the primary degradation product of SAM in case of transfer reactions of the aminocarboxypropyl moiety on uridines in the RNA macromolecules. Furthermore it is built by transfer of propylamino groups on the polyamine compounds putrescine and spermidine via the decarboxylated byproduct of SAM, dcSAM. Polyamines are known to be involved in important cell growth and development processes, which thereby also have great impact in tumor genesis [33]. The tumor-associated, deregulated influence on the metabolic flow of the methionine/polyamine cycle probably leads to an accumulation of MTA. Due to its well-known inhibitory effects on methyltransfer reactions, a simultaneous elevated excretion might take place in tumor genesis [30]. Due to the contrarily proceeding SAM-induced methyltransfer reactions leading to m^6^_1_A and m^6^t^6^A, shifts in the metabolite ratios involving MTA were observed.

Another interesting metabolite ratio is No. 28, featuring cytidine and its acetylated derivative ac^4^C. The latter is built in rRNA and tRNA by means of an acetyltransferase system and most probably acetyl-CoA as donor of the acetyl function [4].

In eukaryotic tRNA, ac^4^C is exclusively implemented on position 12 in the D-loop. The exact biological function is still unknown. A general stabilization of the tRNA structure has been discussed in [34]. Elevated amounts of acetylated cytidine have been described in numerous reports, dealing with the altered excretion of modified nucleosides in cancer diseases [35]. The selection of the C/ac^4^C combination in our classification approach appears in analogy to the results of our previous work with cell culture supernatants, which showed distinctive alterations in the excretion of cytidine in breast cancer cells [30].

Selection of feature combination No. 32 also reflects relevant attributes of tumor-associated alterations of RNA metabolism. The monomethylated guanosine derivative m^2^G and its dimethylated analogon m^2^_2_G derive from eukaryotic tRNA and rRNA [4] and have both been postulated as potential tumor markers [36]. During biosynthesis of the methylated guanosines, the precursor molecule m^2^G is converted to m^2^_2_G via the tRNA-N^2^, N^2^-dimethyltransferase [37]. In tumors of liver and kidney, a distinctively elevated activity of the involved enzyme system has been observed by Craddock [38]. The resulting elevated biosynthesis of m^2^_2_G explains the observed tumor-associated shifts in the m^2^G/m^2^_2_G ratio.

## Conclusion

In conclusion, we found a reasonable set of 44 tumor-related metabolite pairs measured by LC-IT MS with a SVM prediction performance of 83.5% sensitivity and 90.6% specifity (p-value << 0.05). We demonstrate that semiquantitative measurements are valuable for pattern detection using nonparametric machine learning algorithms. Our results constitute the basis for the development of a noninvasive and efficient screening method. Although we have analyzed a balanced dataset of 170 urine samples and estimated the prediction performance using the nearly unbiased LOO, a validation study remains future work. It is essential to perform a large-scale and multi-centric evaluation study of the method to prove it as valid for clinical testing.

## Competing interests

The authors declare that they have no competing interests.

## Authors' contributions

CH performed bioinformatical data analysis. DB performed sample preparation and LC-MS analysis. NF extracted the urinary samples by boronate affinity chromatography. RF, HS, HN as well as CG designed the concept of the clinical study. SL, MS, AZ and BK supervised the study and critically revised the manuscript. All authors read and approved the final manuscript.

## Pre-publication history

The pre-publication history for this paper can be accessed here:

http://www.biomedcentral.com/1471-2407/9/104/prepub

## Supplementary Material

Additional file 1 **Note on the arctan encoding**. This additional note contains more information about the ideas of using the arctan function for encoding pairwise relations. Click here for file

Additional file 2 **Phenotype permutation test**. This table contains p-values for a phenotype permutation test performed for the arctan encoded pairwise features. Click here for file

Additional file 3 **Metabolite variability**. This document contains a boxplot and a discussion of the value codomain for each measured metabolite and collective, e.g. patient and control. Click here for file
