# Supplementary material for: Prediction of breast cancer by profiling of urinary RNA metabolites using Support Vector Machine-based feature selection
Source: BMC Cancer. 2009 Apr 5;9:104. doi: 10.1186/1471-2407-9-104 (PMC2680413; doi:10.1186/1471-2407-9-104)
Supplement: Additional file 1 — Note on the arctan encoding. This additional note contains more information about the ideas of using the arctan function for encoding pairwise relations. [file 1471-2407-9-104-S1.pdf]

Supplementary note for "Prediction of breast cancer by profiling of urinary RNA metabolites using Support Vector Machine-based feature selection"

Carsten Henneges Dino Bullinger Richard Fux
Natascha Friese Harald Seeger Hans Neubauer
Stefan Laufer Gleiter, H.,Christoph Matthias Schwab
Andreas Zell and Bernd Kammerer

February 24, 2009

This supplementary note dicusses the arctan-encoding in more detail. It explains the ideas related to this kind of encoding and provides information and references on the subject of *feature construction.* Finally it provides additional prediction results that were not included in the manuscript.
The first step in our bioinformatical data analysis is to construct appropriate features for training a learning algorithm, for instance the SVM. The standard initial approach is to train on the numerical descriptors that are already given. In our case we first trained models on the semi-quantitative concentrations as a baseline (table 2).
After that we tried to improve our predictions using *feature construction* and *feature selection* according to [2, 3]. It was our major goal to rely on methods that do not introduce assumptions into the approach. We therefore excluded Prinicipal Component Analysis for feature construction from our approach, which comes with the following assumptions (made within its deriviation):

1. Assumption of Linearity
2. Assumption that mean and covariance are statistically important
3. Assumption that large deviations are important.

The next best method without assumptions is to guess a feature construction function. Therefore we experimented with the idea that features are related to each other and that a pairwise encoding is more appropriate for this learning task. Furthermore we thought that features have to be considered relative to each other since they could be substrates or products of each other.
Thus we aimed to encode the relation x/y for concentrations x and y. Clearly

both values of x and y are positive but could be zero. While $x = 0$ is a well defined value, the problematic case arises when $y = 0$. In principle this case could be defined as ∞, but a better encoding for x/y is required since ∞ is surely not adequate for a numerical learning algorithm.
A function that transforms the relation x/y consistently on a finite scale without loss of information is the $\arctan(x, y)$. Therefore we encoded each measured concentration by

$$e(x, y) = \begin{cases} \arctan(x/y) & y \neq 0 \\ \pi/2 & y = 0 \end{cases} \tag{1}$$

using $\lim_{y \to 0} \arctan(x/y) = \pi/2$ as limiting value for the case were $y = 0$. The resulting mapping is shown in figure . As can be seen the codomain is consis-

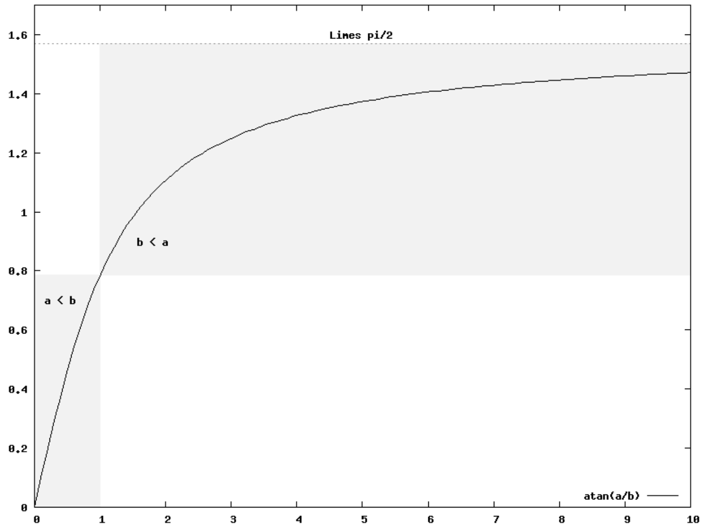

Figure 1: The arctan function maps the whole domain of x/y for positive x, y onto the finite codomain $[0, \pi/2)$. An consistent extension is therefore the definition for $y = 0$ to be the value $\pi/2$ such that the codomain is $[0, \pi/2]$.

tently extended to be $[0, \pi/2]$ for the case when y was not detected, e.g. when metabolites fell below the detection threshold. This occurs a total number of 301 times of $54 \cdot 160 = 8640$ samples being 0.03483% of all values. Consequently this case is not considered to have a major impact for learning. Another beneficial side effect is the small codomain that is supporting the SVM, which is known to perform better when data is scaled to fixed ranges. Thus subsequent scaling or normalization is not required, when using the arctan. Also the information which value was greater is encoded by the arctan (and the fraction, of course) since both cases are mapped onto different ranges within the codomain.
We tested our encoding twice by training with and without arctan encoding on

the newly detected data set as well as on the data set published in [1]. Both times we employed the OSAF on the given data set and determined the best performing feature combination together with its generalization error (results given in table 2). First, comparing the results of the OSAF-SVM combination with the previous published performance of the SVM in [1] reveals that sensitivity is increased for the LOO from 74% to 83% and specificity is increased from 85% to 88%, when training on the simple concentrations. Using the arctan encoding also improves this performance to 89% sensitivity and 91% specificity. Evaluating the OSAF-SVM on our data set without arctan encoding yields a sensitivity of 77% and a specificity of 69% for the LOO. Using the arctan encoding improves this to 84% sensitivity and 86% specificity. Therefore in both cases the arctan encoding improved the prediction performance of the OSAF-SVM combination.
In summary, we have compared our approach to the case of no normalization and found that a pairwise encoding of the features mapped onto a limited codomain supports the generation of predictive SVM models.

Data set	Encoding	Validation	MCC	Sensitivity	Specificity
Previous [1]	simple	10fold CV	76.7208	0.849558	0.919192
	simple	LOO	71.9206	0.831858	0.888889
Previous [1]	arctan	10fold CV	85.8483	0.920354	0.939394
	arctan	LOO	80.1715	0.893805	0.909091
Manuscript	simple	10fold CV	51.9411	0.800000	0.717647
	simple	LOO	47.2192	0.776471	0.694118
Manuscript	arctan	10fold CV	74.303	0.835294	0.905882
	arctan	LOO	69.431	0.835294	0.858824

Figure 2: This table shows the results of comparing the arctan encoding on two different data set.

References

[1] Dino Bullinger, Holger Fröhlich, Fabian Klaus, Hans Neubauer, Antje Frickenschmidt, Carsten Henneges, Andreas Zell, Stefan Laufer, Christoph H. Gleiter, Hartmut Liebich, and Bernd Kammerer. Bioinformatical evaluation of modified nucleosides as biomedical markers in diagnosis of breast cancer. *Analytica Chimica Acta*, 618(1):29 – 34, 2008.

[2] I. Guyon and A. Elisseeff. An introduction to variable and feature selection. *The Journal of Machine Learning Research*, 3:1157–1182, 2003.

[3] P. Somol and P. Pudil. Oscillating Search Algorithms for Feature Selection. In *International Conference on Pattern Recognition*, volume 15, pages 406–409, 2000.
