# Supplementary material for: Prediction of breast cancer by profiling of urinary RNA metabolites using Support Vector Machine-based feature selection
Source: BMC Cancer. 2009 Apr 5;9:104. doi: 10.1186/1471-2407-9-104 (PMC2680413; doi:10.1186/1471-2407-9-104)
Supplement: Additional file 3 — Metabolite variability. This document contains a boxplot and a discussion of the value codomain for each measured metabolite and collective, e.g. patient and control. [file 1471-2407-9-104-S3.doc]

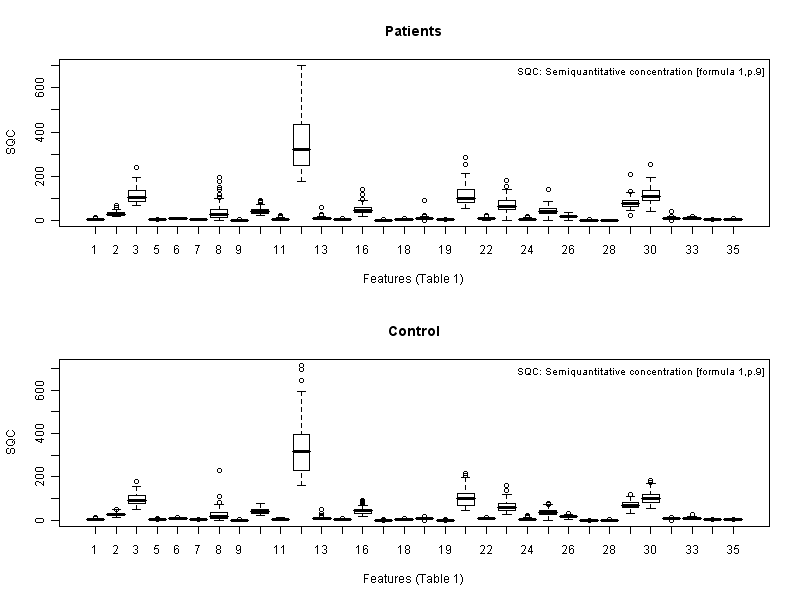


This plot shows that the behaviour of the variability correlates very well between both collectives. For example inspect feature 20. The domain of its values is nearly the same in both collectives. In the patients collective the codomain ranges from approx. 120 to 180, and in the control collective from approx. 100 to 150. Thus both ranges overlap very well and the degree of its variability is nearly the same in both collectives. This is also true for the other features. Furthermore we find that the variability is negligible for 23 of the 35 features. But there exist also differences. For instance, the median of feature 20 is lower for the patients’ collective than for the control. This indicates a slight shift in the metabolism of 22, which is subsequently incorporated into the arctan features during encoding and then exploited when training the SVM. In conclusion, the discrimination of patients and controls can not be based on different value domains, but has to rely on altered metabolism within given value ranges.
